# Comprehensive Echocardiographic Assessment of Right Ventricular Afterload and Right Ventricular–Pulmonary Arterial Coupling in the Intensive Care Unit

**DOI:** 10.1155/ccrp/3346063

**Published:** 2026-10-28

**Authors:** Aibek E. Mirrakhimov

**Affiliations:** ^1^ Department of Anesthesiology, Perioperative, Critical Care, and Pain Medicine Division of Adult Cardiothoracic Anesthesiology University of Kentucky Lexington Kentucky USA uky.edu

## Abstract

Right ventricular (RV) dysfunction is common in critical illness, yet RV afterload cannot be adequately characterized by pulmonary artery systolic pressure (PASP) alone. Bedside echocardiography can estimate right atrial and pulmonary artery pressures, suggest increased pulmonary vascular resistance, and assess RV–pulmonary arterial coupling while simultaneously evaluating RV size, systolic function, septal interaction, and forward flow. Peak tricuspid regurgitation velocity combined with right atrial pressure estimates RV systolic pressure and, in the absence of outflow obstruction, approximates PASP. Pulmonary regurgitation velocities provide complementary estimates of mean and end‐diastolic pulmonary artery pressure, whereas pulmonary artery acceleration time, RV outflow tract velocity–time integral, and systolic notching provide information regarding pulmonary vascular load and forward flow. A multiparametric assessment of RV systolic performance is essential because tricuspid annular plane systolic excursion, tissue Doppler systolic velocity, fractional area change, RV free‐wall strain, and three‐dimensional RV ejection fraction assess different aspects of contraction. The tricuspid annular plane systolic excursion/PASP ratio relates longitudinal systolic performance to pulmonary pressure but remains a surrogate of coupling, without a universal intensive care threshold. These measurements depend on image quality, loading conditions, and ventilatory support and may differ substantially from invasive hemodynamics in individual patients. Their greatest value lies in integrated physiologic assessment and serial reassessment after treatment. Echocardiography complements right heart catheterization, which remains necessary when precise hemodynamic classification or measurement of pulmonary artery wedge pressure, cardiac output, or pulmonary vascular resistance will change management.

## 1. Introduction

Acute right ventricular (RV) dysfunction may complicate pulmonary embolism, acute respiratory distress syndrome (ARDS), hypoxemia, hypercapnia, sepsis, decompensated pulmonary hypertension, left‐sided heart failure, cardiac surgery, and positive‐pressure ventilation, among other conditions [1]. In these settings, bedside echocardiography is valuable because it can be easily repeated after changes in ventilation, vasoactive therapies, volume status, or pulmonary vasodilator treatment. A single estimated pulmonary artery systolic pressure (PASP), however, is an incomplete description of RV loading.

RV afterload comprises a steady component, represented primarily by pulmonary vascular resistance (PVR), and pulsatile components that include pulmonary arterial compliance, characteristic impedance, and wave reflection [2]. Pulmonary artery pressure is also influenced by pulmonary artery wedge pressure (PAWP), also termed pulmonary artery occlusion pressure, cardiac output, and RV contractile capacity. Consequently, an elevated PASP may reflect increased left‐sided filling pressure, increased pulmonary blood flow, elevated PVR, or a combination of these mechanisms. Conversely, a severely failing RV may generate only modest pulmonary artery pressures despite a critically increased pulmonary vascular load. A comprehensive intensive care assessment should therefore integrate pressure estimates, forward flow, pulmonary artery waveform morphology, and RV performance relative to afterload.

In this article, we examine RV afterload as a pressure–flow relationship and emphasize that RV function cannot be understood independently of the load against which the ventricle ejects. We first discuss right ventricular–pulmonary arterial (RV–PA) coupling and image acquisition, followed by assessment of RV systolic function, right‐sided pressures, and PVR. We then present a practical integrated approach and discuss common phenotypes of RV dysfunction in the intensive care unit (ICU).

## 2. Physiologic Framework: RV Afterload and RV–PA Coupling

The pulmonary circulation normally provides a low‐resistance, highly compliant circuit, allowing the thin‐walled RV to eject a large stroke volume against minimal afterload [2]. The normal RV is similarly compliant and can accommodate a larger end‐diastolic volume than the left ventricle (LV) without a substantial increase in filling pressure. In critical illness, PVR may increase because of hypoxemia, hypercapnia, acidemia, pulmonary embolism, preexisting pulmonary hypertension, or excessive lung inflation from high tidal volumes or positive end‐expiratory pressure (PEEP) [3]. PVR is lowest near functional residual capacity and increases at both lower and higher lung volumes. At low lung volumes, reduced radial traction narrows the extra‐alveolar vessels, whereas at high lung volumes, alveolar expansion compresses the alveolar capillaries. These mechanisms are summarized in Figure 1.

**FIGURE 1:**
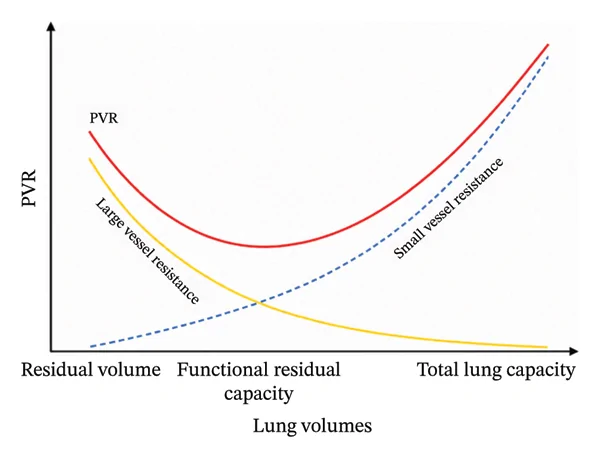
Relationship between lung volume and pulmonary vascular resistance (PVR). PVR is lowest near functional residual capacity. At low lung volumes, reduced radial traction increases extra‐alveolar vessel resistance, whereas at high lung volumes, alveolar expansion compresses the alveolar capillaries. The curves labeled large‐vessel and small‐vessel resistance represent these extra‐alveolar and alveolar contributions, respectively. Consequently, total PVR follows a U‐shaped relationship with lung volume.

RV afterload, however, depends not only on PVR, which opposes steady forward flow but also on pulmonary arterial stiffness, which increases the pressure required for pulsatile ejection. Pressure waves generated by RV ejection are reflected from distal vascular branch points and areas of increased impedance. When the pulmonary arteries become stiff, these waves travel faster and return earlier during systole, while the RV is still ejecting, thereby further increasing RV afterload. Because PASP does not distinguish between these components, it cannot fully characterize the total load faced by the RV.

The invasive reference standard for RV–PA coupling is the ratio of RV end‐systolic elastance to effective arterial elastance (Ees/Ea), derived from pressure–volume analysis [2]. Ees describes RV contractility, whereas Ea provides a lumped measure of the load against which the RV ejects. Coupling is preserved when RV contractility increases sufficiently to accommodate the rising afterload while maintaining efficient energy transfer. Uncoupling occurs when this compensatory response becomes inadequate, leading to progressive RV dilation, increased wall stress, tricuspid regurgitation, reduced forward flow, exaggerated ventricular interdependence, and systemic venous congestion. The physiology of RV–PA coupling is summarized in Figure 2.

**FIGURE 2:**
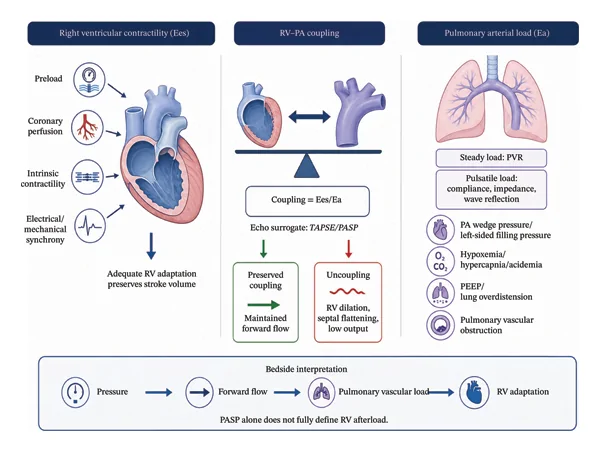
Overview of right ventricular–pulmonary arterial (RV–PA) coupling. Coupling is preserved when RV contractility increases sufficiently to match the prevailing afterload. Progressive mismatch between contractility and afterload leads to RV dilation, increased wall stress, tricuspid regurgitation, reduced forward flow, ventricular interdependence, and systemic venous congestion. The left panel summarizes factors affecting overall RV performance; preload is not a measure of intrinsic contractility. Abbreviations: Ea, effective arterial elastance; Ees, RV end‐systolic elastance; PA, pulmonary artery; PASP, pulmonary artery systolic pressure; PEEP, positive end‐expiratory pressure; PVR, pulmonary vascular resistance; TAPSE, tricuspid annular plane systolic excursion.

### 2.1. Technical Conditions and Acquisition

Hemodynamic and ventilatory conditions should be documented at the time of imaging, including heart rate, rhythm, systemic blood pressure, ventilatory mode, PEEP, oxygenation, and vasoactive or pulmonary vasodilator support. Comparisons with pulmonary artery catheterization are most meaningful when measurements are obtained contemporaneously and under similar physiologic conditions.

A comprehensive transthoracic echocardiographic (TTE) examination should include parasternal RV inflow and outflow, parasternal short‐axis at the midpapillary level, RV‐focused apical four‐chamber, and subcostal views, with additional assessment of the inferior vena cava (IVC) (Figure 3A–F) [4, 5]. Tricuspid regurgitation (TR) should be interrogated from multiple windows to identify the best‐aligned Doppler signal. In patients with limited TTE windows, transesophageal echocardiography (TEE) may provide complementary information when clinically appropriate (Figure 4A–F). Useful TEE views include the mid‐esophageal RV‐focused four‐chamber, RV inflow–outflow, ascending aortic short‐axis, modified bicaval, upper‐esophageal aortic arch short‐axis, and transgastric midpapillary short‐axis views. Leftward interventricular septal flattening producing a D‐shaped LV suggests RV pressure or volume overload: flattening that is most prominent at end‐systole favors pressure overload (Figure 3C), whereas predominant end‐diastolic flattening favors volume overload [6].

**FIGURE 3:**
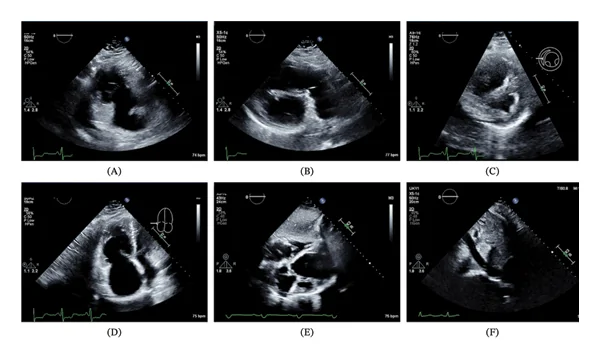
Transthoracic echocardiographic views for assessment of RV size, function, and loading conditions. (A) Parasternal RV inflow view. (B) Parasternal RV outflow view. (C) Parasternal short‐axis view at the midpapillary level demonstrating end‐systolic septal flattening and a D‐shaped left ventricle, consistent with RV pressure overload. (D) RV‐focused apical four‐chamber view. (E) Subcostal four‐chamber view. (F) Long‐axis view of the inferior vena cava.

**FIGURE 4:**
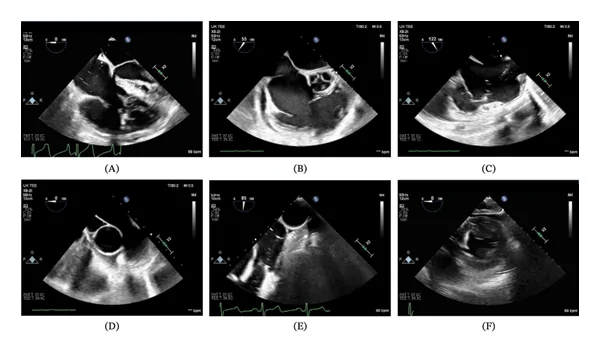
Transesophageal echocardiographic views for assessment of RV size, function, and loading conditions. (A) Mid‐esophageal RV‐focused four‐chamber view. (B) Mid‐esophageal RV inflow–outflow view. (C) Mid‐esophageal modified bicaval view. (D) Mid‐esophageal ascending aortic short‐axis view. (E) Upper‐esophageal aortic arch short‐axis view. (F) Transgastric short‐axis view at the midpapillary level.

The Doppler beam should be aligned as parallel as possible to the regurgitant jet. Peak TR velocity should be measured at the dense outer edge of a complete continuous‐wave Doppler envelope, excluding faint feathering or duplicated artifacts [4]. Incomplete envelopes may underestimate velocity, whereas excessive gain, spectral blooming, or tracing a faint duplicated envelope may lead to overestimation. Multiple representative beats should be averaged, particularly in atrial fibrillation or when substantial respiratory variation is present.

### 2.2. Respiratory Pressure and Transmural Load

Positive‐pressure ventilation has profound effects on RV preload and afterload [7, 8]. Doppler‐derived pulmonary pressure estimates and invasive catheter measurements represent intraluminal pressures, whereas RV distending pressure is transmural, defined as intracavitary pressure minus surrounding pericardial pressure. Pleural pressure influences this surrounding pressure, although the two are not always equivalent. High airway and pleural pressures may raise measured right atrial and pulmonary artery pressures without an equivalent increase in transmural distending pressure. Simultaneously, as illustrated in Figure 1, lung overdistension, hypoxemia, hypercapnia, and acidemia may increase PVR, while reduced venous return and RV stroke volume may lower a Doppler‐derived pressure gradient. Therefore, a change in estimated PASP after ventilator adjustment cannot be interpreted in isolation. These effects have been demonstrated in physiologic studies of PEEP, hypercapnia, and lung recruitability [9–11].

For serial assessment, measurements should be obtained at a reproducible respiratory phase and, when feasible, at similar PEEP and ventilatory support. Changes in pressure should be interpreted alongside RV outflow tract (RVOT) velocity–time integral (VTI), RV geometry, septal configuration, systemic perfusion, and multiparametric RV systolic performance.

### 2.3. RV Contraction Pattern and Echocardiographic Assessment of Systolic Function

RV contraction differs substantially from LV contraction. The LV has a relatively symmetric ellipsoidal geometry and ejects through coordinated longitudinal shortening, circumferential and radial contraction, and torsion. In contrast, the thin‐walled, crescent‐shaped RV contracts through longitudinal shortening from base to apex, inward radial displacement of the RV free wall (the bellows effect), and anteroposterior shortening related partly to LV traction and ventricular interdependence. LV contraction also contributes to RV ejection through interventricular septal contraction and mechanical traction transmitted across shared myocardial fibers. The complex anatomy and contraction pattern of the RV complicate its echocardiographic assessment. Three‐dimensional echocardiography demonstrates that nonlongitudinal motion contributes substantially to normal global RV ejection [12, 13].

The contraction pattern changes with disease and increasing afterload. In pulmonary hypertension, longitudinal shortening may deteriorate relatively early while radial and anteroposterior components initially compensate. With progressive RV–PA uncoupling, this compensation is lost. Rako et al. found that reduced longitudinal shortening was associated with earlier uncoupling, whereas impaired anteroposterior shortening characterized more advanced uncoupling and was accompanied by reduced LV preload [14]. Individual echocardiographic indices therefore interrogate different components of RV contraction and should not be treated as interchangeable measurements of a single property.

### 2.4. Conventional and Advanced Echocardiographic Measures

Unless otherwise specified, the reference limits in this subsection follow the 2025 American Society of Echocardiography recommendations and are predominantly derived from TTE populations [4]. They are reference values for interpretation, not treatment targets validated for critically ill patients.

RV size should be assessed both qualitatively and quantitatively at end‐diastole, preferably from a nonforeshortened RV‐focused apical four‐chamber TTE view or, when TTE imaging is inadequate or unavailable, from an RV‐focused mid‐esophageal four‐chamber TEE view. Qualitatively, the normal RV should appear to be no more than approximately two‐thirds of the LV size, with the LV apex remaining predominant. Progressive loss of LV predominance, approximate equality of RV and LV dimensions, or an RV larger than the LV supports increasing RV dilation (Figure 5). However, this relative comparison may be misleading when the LV is itself dilated or underfilled. Quantitative assessment includes the basal, midventricular, and longitudinal RV dimensions, with normal values of < 4.1 cm, < 3.5 cm, and < 8.2 cm, respectively. The normal RV end‐diastolic area is < 25 cm^2^ or < 14 cm^2^/m^2^ when indexed to the body surface area. When image quality and software permit, three‐dimensional RV volumes provide a more comprehensive assessment of chamber size. An RV‐focused deep transgastric TEE view may provide additional information in selected patients when visualization from the mid‐esophageal view is inadequate. However, most normative values and grading thresholds are derived from TTE and should not be assumed to be directly interchangeable with TEE measurements. Representative RV end‐diastolic dimensions obtained with TEE are shown in Figure 6.

**FIGURE 5:**
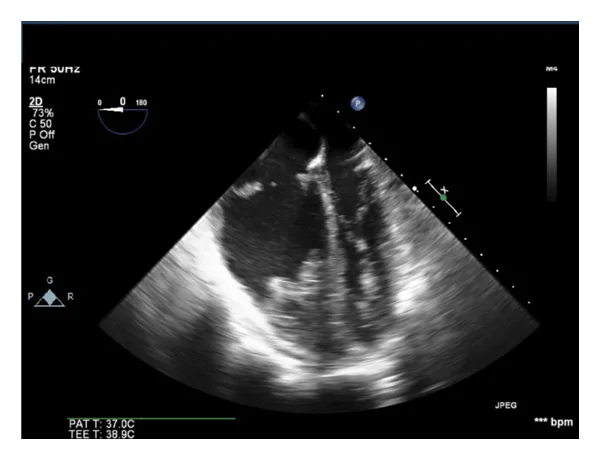
RV‐focused mid‐esophageal four‐chamber transesophageal echocardiographic view demonstrating marked RV enlargement and an underfilled left ventricle in a patient with massive pulmonary embolism and tricuspid valve infective endocarditis.

**FIGURE 6:**
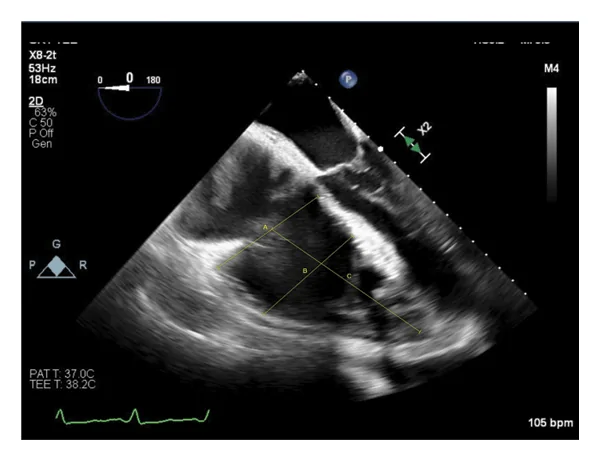
RV‐focused mid‐esophageal four‐chamber transesophageal echocardiographic view at end‐diastole demonstrating a basal RV diameter of 6.8 cm, midventricular diameter of 6.4 cm, and longitudinal diameter of 9.6 cm, consistent with marked RV enlargement.

RV free‐wall thickness should be measured at end‐diastole, preferably from the subcostal four‐chamber TTE view at the level of the RV inflow, between the tricuspid annulus and papillary muscles. Trabeculations, papillary muscles, and pericardial fat should be excluded from the measurement. RV wall thickness may also be assessed from an RV‐focused apical four‐chamber TTE view or an RV‐focused mid‐esophageal four‐chamber TEE view when the endocardial and epicardial borders are clearly visualized and the ultrasound beam is appropriately aligned, although these approaches are less standardized. A free‐wall thickness ≥ 0.5 cm is abnormal and supports the presence of right ventricular hypertrophy (RVH). In the ICU, RVH suggests chronic pressure loading or chronic RV remodeling and may help distinguish preexisting pulmonary hypertension from an acute increase in RV afterload. However, RVH is nonspecific and may also occur in inherited or infiltrative cardiomyopathies. An example of RVH is shown in Figure 7.

**FIGURE 7:**
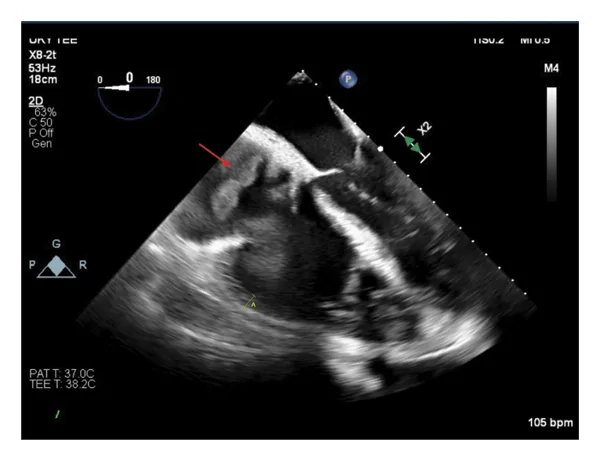
RV‐focused mid‐esophageal four‐chamber transesophageal echocardiographic view at end‐diastole demonstrating RV free‐wall thickness of 8 mm in a patient with massive pulmonary embolism and preexisting group 2 pulmonary hypertension due to left‐sided heart disease. A large right atrial thrombus is present (red arrow), consistent with thrombus in transit.

Tricuspid annular plane systolic excursion (TAPSE) measures the systolic longitudinal displacement of the lateral tricuspid annulus toward the apex and predominantly reflects the basal longitudinal RV function. A TAPSE > 1.7 cm is considered normal. Although simple and reproducible, TAPSE is one‐dimensional, load‐dependent, and influenced by cardiac translation, severe TR, pacing, regional disease, and altered RV geometry. Following cardiac surgery, longitudinal annular motion commonly decreases despite the relatively preserved global RV systolic function [5]. Significant TR further complicates interpretation because annular motion and forward output may become discordant [15].

TAPSE is typically measured using M‐mode echocardiography by placing the cursor through the lateral tricuspid annulus and aligning it parallel to annular motion in the RV‐focused apical four‐chamber TTE view (Figure 8). It may also be measured from the RV‐focused mid‐esophageal four‐chamber TEE view using angle‐corrected anatomic M‐mode (Figure 9). Angle correction is often required because the fixed position of the esophageal probe may prevent the conventional M‐mode cursor from aligning with longitudinal annular motion. However, angle‐corrected M‐mode remains dependent on the accurate identification of the annulus and its direction of travel and cannot compensate for out‐of‐plane motion. Residual misalignment may therefore underestimate TAPSE.

**FIGURE 8:**
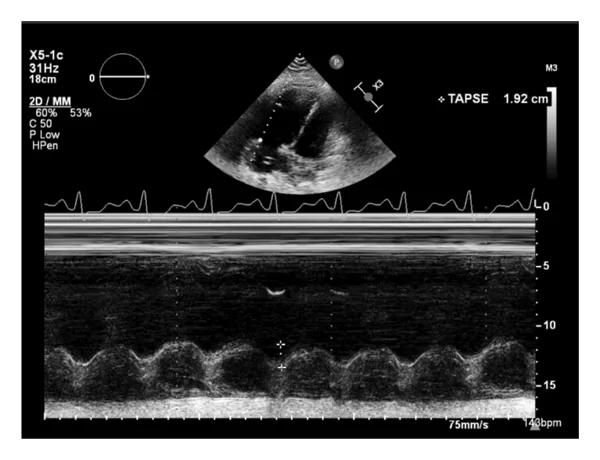
M‐mode measurement of tricuspid annular plane systolic excursion from an RV‐focused apical four‐chamber transthoracic echocardiographic view, demonstrating a value of 1.92 cm.

**FIGURE 9:**
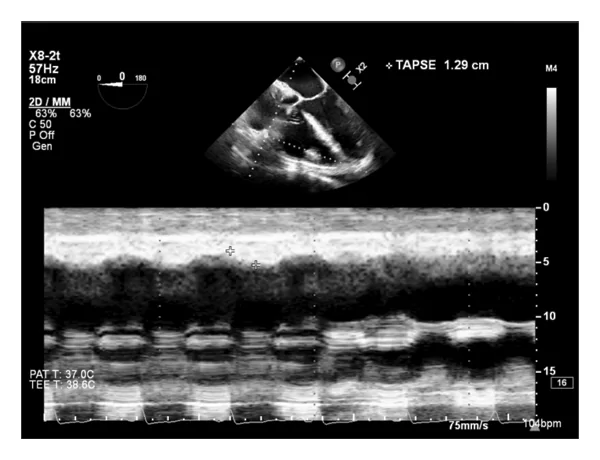
Angle‐corrected anatomic M‐mode measurement of tricuspid annular plane systolic excursion from an RV‐focused mid‐esophageal four‐chamber transesophageal echocardiographic view, demonstrating a value of 1.29 cm.

Tissue Doppler systolic velocity (S′) measures the peak systolic myocardial velocity at the lateral tricuspid annulus. A value > 9.5 cm/s is considered normal when obtained from the standard RV‐focused apical four‐chamber TTE view. Like TAPSE, S′ primarily reflects basal longitudinal contraction. However, TAPSE measures the total distance traveled by the tricuspid annulus during systole, whereas S′ measures its peak instantaneous velocity. Because velocity represents the rate of displacement, the two measurements may diverge when the timing or duration of systolic shortening differs. Tissue Doppler capability is required and may not be available on every portable or limited‐function ultrasound system in the ICU.

On TTE, S′ is typically acquired from the RV‐focused apical four‐chamber view (Figure 10). With TEE, alignment with longitudinal annular motion may be achieved from the transgastric RV inflow–outflow view (Figure 11) or, in selected patients, from an RV‐focused deep transgastric view. The deep transgastric approach requires greater probe advancement and manipulation and should be reserved for situations in which standard views are nondiagnostic and the information is expected to influence patient management. S′ has excellent temporal resolution but is angle‐dependent and samples only a single basal region. It may therefore be misleading after cardiac surgery or whenever basal longitudinal motion is discordant with the global RV function. TEE‐derived S′ should also be interpreted cautiously because reference values established using TTE cannot be applied directly to transgastric TEE measurements.

**FIGURE 10:**
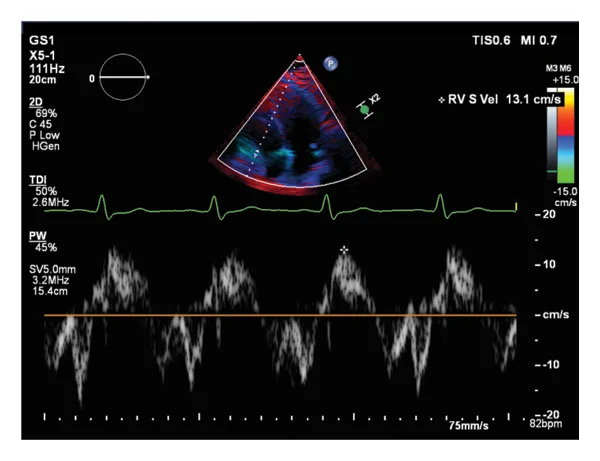
Tissue Doppler measurement of lateral tricuspid annular systolic velocity (S′) from an RV‐focused apical four‐chamber transthoracic echocardiographic view, demonstrating a value of 13.1 cm/s.

**FIGURE 11:**
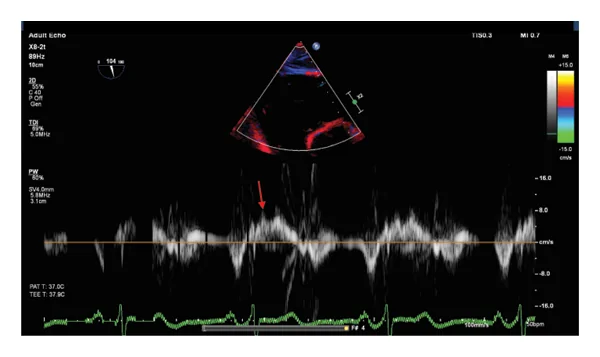
Tissue Doppler measurement of lateral tricuspid annular systolic velocity (S′) from a transgastric right ventricular inflow–outflow transesophageal echocardiographic view. The red arrow identifies the systolic tissue Doppler signal.

RV fractional area change (FAC) is calculated as [(RV end‐diastolic area − RV end‐systolic area)/RV end‐diastolic area] × 100. An FAC > 35% is considered normal. FAC is typically measured from an RV‐focused apical four‐chamber TTE view or an RV‐focused mid‐esophageal four‐chamber TEE view (Figure 12), provided that the entire RV cavity and endocardial border are adequately visualized. Unlike TAPSE or S′, FAC incorporates longitudinal shortening, inward free‐wall motion, and septal contribution within the four‐chamber plane, providing a more global two‐dimensional assessment of RV systolic function. However, its accuracy depends on obtaining a nonforeshortened view, correctly identifying end‐diastole and end‐systole, and accurately tracing the endocardial border. Poor image quality and prominent trabeculations may make border delineation difficult and increase measurement variability. FAC also excludes the RV outflow tract and reduces a complex three‐dimensional chamber to a single imaging plane. Measurement requires area‐planimetry capability, which may not be available on all portable or limited‐function ultrasound systems used in the ICU.

**FIGURE 12:**
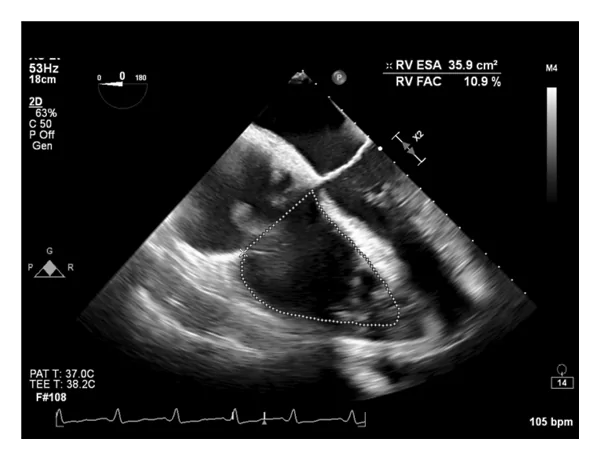
RV‐focused mid‐esophageal four‐chamber transesophageal echocardiographic view used for measurement of RV fractional area change. The displayed end‐systolic tracing and calculated fractional area change of 10.9% demonstrate markedly reduced RV systolic function. Fractional area change incorporates longitudinal and inward free‐wall motion within a two‐dimensional plane.

Speckle‐tracking‐derived RV longitudinal strain quantifies myocardial deformation rather than annular displacement. Unlike tissue Doppler, speckle tracking is relatively angle‐independent because it follows acoustic markers within the two‐dimensional imaging plane, although it cannot account for out‐of‐plane motion. RV global longitudinal strain includes both the interventricular septum and RV free wall, whereas RV free‐wall strain averages the basal, mid‐, and apical free‐wall segments and excludes the septal segments. Nevertheless, free‐wall deformation remains influenced by ventricular interdependence. Because longitudinal shortening produces negative strain, less negative values indicate reduced myocardial deformation. RV free‐wall strain values more negative than approximately −20% are generally considered normal, although reference limits vary according to software, vendor, age, sex, and population. RV free‐wall strain may identify dysfunction despite preserved conventional indices but remains load‐dependent; increased afterload may reduce strain without necessarily indicating impaired intrinsic contractility. For TTE, strain is typically acquired from a nonforeshortened RV‐focused apical four‐chamber view with adequate visualization of the entire RV free wall. An RV‐focused mid‐esophageal four‐chamber TEE view (Figure 13) may be used when TTE imaging is unavailable or inadequate. Dedicated strain‐analysis software and satisfactory myocardial tracking are required and may not be available on all ultrasound systems used in the ICU. Moreover, because most reference values are derived from TTE, TEE‐derived measurements should be interpreted with caution.

**FIGURE 13:**
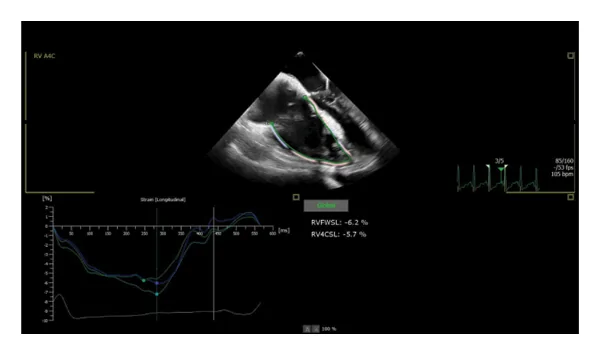
Speckle‐tracking analysis of RV longitudinal strain from an RV‐focused mid‐esophageal four‐chamber transesophageal echocardiographic view. The displayed RV free‐wall strain of −6.2% and four‐chamber strain of −5.7% indicate markedly reduced longitudinal deformation. RVFWSL, RV free‐wall longitudinal strain; RV4CSL, RV four‐chamber longitudinal strain.

RV dP/dt reflects the rate of RV pressure rise during early systole and serves as an adjunctive measure of RV systolic performance. It is calculated from the ascending limb of the TR continuous‐wave Doppler envelope by measuring the time required for TR velocity to increase from 1 to 2 m/s. Using the modified Bernoulli equation, this change corresponds to an increase in the RV‐to‐right atrial (RA) pressure gradient from 4 to 16 mmHg, or 12 mmHg:

(1) RV dPdtmmHg/s=12Δts=12000,Δtms.

Here, Δt is the time required for TR velocity to increase from 1 to 2 m/s. A value < 400 mmHg/s is considered abnormal, although normative data are limited and this measurement is not recommended for routine use.

Measurement requires a definite TR jet, a dense early systolic envelope recorded at a high sweep speed, preferably approximately 200 mm/s, and Doppler alignment parallel to the TR jet. The jet should be interrogated from RV‐focused apical four‐chamber or parasternal RV inflow TTE views, or mid‐esophageal RV inflow–outflow or modified bicaval TEE views, using the best‐aligned signal (Figure 14). RV dP/dt is unavailable without TR and unreliable with incomplete or misaligned envelopes. It is load‐dependent, assumes relatively stable RA pressure, and may be affected by severe TR, heart rate, rhythm, and positive‐pressure ventilation. It should therefore complement rather than replace multiparametric assessment of RV systolic function.

**FIGURE 14:**
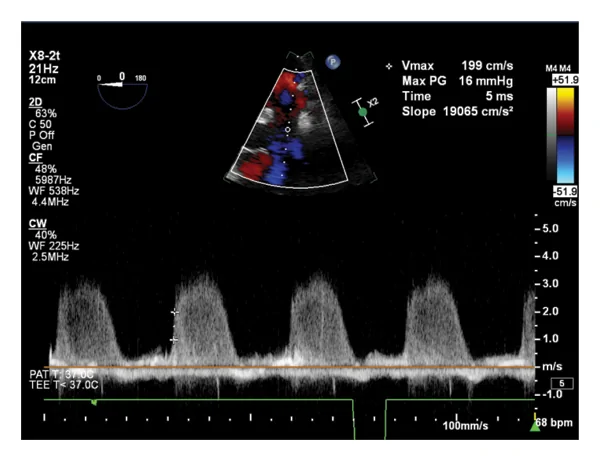
Transesophageal echocardiographic measurement of the rate of right ventricular pressure rise (RV dP/dt). An increase in tricuspid regurgitation velocity from 1 to 2 m/s over 5 ms corresponds to RV dP/dt of 12,000/5 = 2400 mmHg/s. The displayed example was recorded at 100 mm/s; a higher sweep speed is preferable when measuring very short intervals.

Three‐dimensional echocardiographic right ventricular ejection fraction (RVEF) integrates longitudinal, radial, and anteroposterior components of RV contraction without relying on the geometric assumptions inherent to two‐dimensional imaging (Figure 15). An RVEF > 45% is considered normal. Three‐dimensional echocardiography also provides RV end‐diastolic and end‐systolic volumes and allows a more comprehensive volumetric assessment when the entire RV is adequately captured [16]. Cardiac magnetic resonance imaging remains the reference standard for RV volume and ejection fraction assessment. Limitations of three‐dimensional echocardiography include incomplete visualization of the entire RV, lower temporal and spatial resolution, stitching artifacts with multibeat acquisition, uncertainty in endocardial border delineation, and dependence on vendor‐specific software. The technique also requires dedicated equipment and technical expertise and remains of limited availability, particularly in the ICU.

**FIGURE 15:**
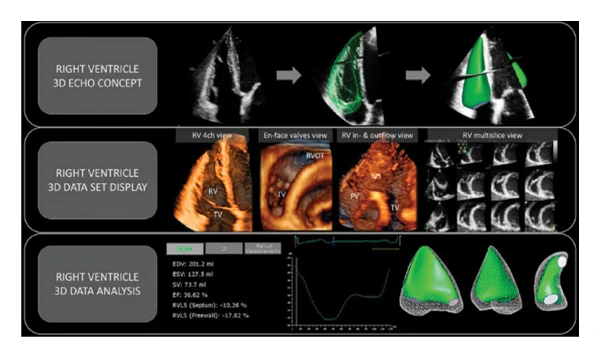
Three‐dimensional echocardiographic assessment of the RV. Upper panel: a three‐dimensional dataset incorporates RV components that may fall outside a single two‐dimensional imaging plane. Middle panel: volume rendering and multiplanar displays permit assessment of RV morphology, valves, outflow tract, septum, and regional motion. Lower panel: dedicated analysis provides RV volumes, ejection fraction, and a volume–time curve. Reproduced from Figure 2 in Surkova et al., “The dysfunctional right ventricle: the importance of multi‐modality imaging” [16], under the Creative Commons Attribution 4.0 International license (https://creativecommons.org/licenses/by/4.0/). Abbreviations within the image: 2D, two‐dimensional; 3D, three‐dimensional; 4ch, four‐chamber; EDV, end‐diastolic volume; EF, ejection fraction; ESV, end‐systolic volume; IVS, interventricular septum; PV, pulmonic valve; RVLS, RV longitudinal strain; RVOT, RV outflow tract; SV, stroke volume; TV, tricuspid valve.

These differences support a multiparametric assessment of RV systolic function. TAPSE and S′ assess basal longitudinal motion, FAC incorporates two‐dimensional longitudinal and inward shortening, RV free‐wall strain measures longitudinal deformation, and three‐dimensional RVEF evaluates global pump performance. Discordance should prompt physiologic interpretation rather than selection of a single “correct” value. Although TEE can provide many of these measurements, most thresholds are TTE‐derived, and differences in imaging planes, Doppler alignment, and software limit interchangeability. Sedation, anesthesia, and positive‐pressure ventilation also alter RV loading conditions and load‐dependent measurements. These considerations are summarized in Table 1.

**TABLE 1:** Selected echocardiographic measures of RV systolic function.

Parameter	Normal value	Major limitations
TAPSE	> 1.7 cm	Load and angle dependence; regional measurement; cardiac surgery, pacing, and severe TR
S′	> 9.5 cm/s	Angle dependence; single basal segment; translation and postoperative changes
FAC	> 35%	Image quality; excludes RVOT; single‐plane geometric limitation
RV free‐wall strain	More negative than −20%	Vendor and population variability; tracking quality; load dependence
Three‐dimensional RVEF	> 45%	Entire‐RV acquisition; temporal resolution; stitching and software dependence

### 2.5. Right Atrial Pressure (RAP)

In spontaneously breathing adults, RAP is commonly estimated from IVC diameter and inspiratory collapse, typically using the subcostal IVC view (Figure 16). An IVC ≤ 2.1 cm with ≥ 50% collapse supports an RAP of approximately 3 mmHg, whereas an IVC > 2.1 cm with < 50% collapse supports an RAP of approximately 15 mmHg. Discordant findings are commonly assigned an RAP of 8 mmHg [4]. The echocardiographic estimation of RAP in spontaneously breathing adults is summarized in Table 2.

**FIGURE 16:**
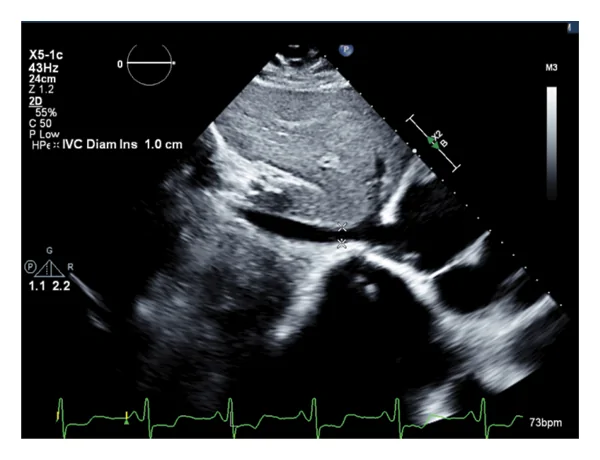
Subcostal long‐axis transthoracic echocardiographic view of the inferior vena cava, demonstrating an inspiratory diameter of 1.0 cm in a spontaneously breathing patient. Diameter should be interpreted with respiratory variation; this still image alone does not establish right atrial pressure.

**TABLE 2:** Estimation of RAP from IVC size and inspiratory collapse in spontaneously breathing adults.

IVC finding	Estimated RAP	Interpretation
≤ 2.1 cm with ≥ 50% inspiratory collapse	Approximately 3 mmHg	Most applicable during spontaneous breathing
Discordant diameter and collapse	Approximately 8 mmHg	Intermediate estimate; seek corroborating evidence
> 2.1 cm with < 50% inspiratory collapse	Approximately 15 mmHg	May indicate elevated RAP during spontaneous breathing

It is important to note that the conventional 3‐, 8‐, and 15‐mmHg categories were not developed as RAP estimates for mechanically ventilated critically ill patients. Positive‐pressure ventilation, high PEEP, spontaneous respiratory effort, elevated intra‐abdominal pressure, obesity, open‐chest conditions, venous cannulae, and severe right‐sided disease may alter IVC diameter and respiratory variation. It is therefore preferable to report the observed IVC findings and their limitations rather than assign unwarranted precision to the estimated RAP. When a reliable central venous or pulmonary artery catheter pressure is available, the measured RAP should generally replace the IVC‐derived estimate in Doppler equations. IVC variation alone should not be interpreted as a measure of fluid responsiveness.

### 2.6. Echocardiographic Estimation of PASP

The modified Bernoulli equation converts peak TR velocity into the estimated systolic pressure gradient between the RV and RA. The approach has been evaluated using simultaneous Doppler and catheter measurements [17]:

(2) RV–RA systolic gradient mmHg=4×VTR2,

(3) RV systolic pressure mmHg=4×VTR2+RAP.

Here, VTR is the peak TR velocity in m/s and RAP is expressed in mmHg. In the absence of pulmonic stenosis, RVOT obstruction, or substantial pressure loss across a conduit, RV systolic pressure approximates PASP. The squared velocity term magnifies acquisition error. At a TR velocity of 4.0 m/s, an error of only 0.2 m/s changes the calculated gradient by approximately 6–7 mmHg before any RAP error is added. An example of the TR velocity measurement by TTE is shown in Figure 17.

**FIGURE 17:**
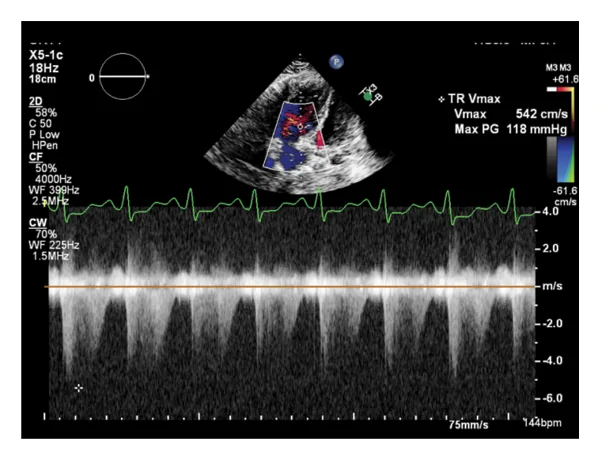
Transthoracic continuous‐wave Doppler recording of tricuspid regurgitation, demonstrating a peak velocity of 5.42 m/s. This corresponds to an RV–RA systolic gradient of approximately 118 mmHg before addition of right atrial pressure. RA, right atrial; RV, right ventricular.

Not every patient with RV dysfunction has a measurable TR jet, and mild TR may produce an incomplete spectral envelope. The absence of a suitable envelope does not exclude pulmonary hypertension. Severe TR may produce an early‐peaking triangular envelope because RV and RA pressures equalize rapidly. In advanced RV failure, reduced stroke volume and impaired contractility may also produce a deceptively low TR velocity. PASP therefore reflects the pressure‐generating capacity of the RV as well as pulmonary vascular load. A low TR velocity in a severely dilated, poorly contracting RV with low RVOT VTI should not be interpreted as reassuring. Conversely, a high PASP or TR velocity in a compensated RV with preserved forward flow does not by itself establish RV failure [15].

Primary validation studies demonstrate substantially better agreement at the population level than precision in individual patients. Fisher et al. and D’Alto et al. demonstrated clinically important overestimation and underestimation of echocardiographically derived pulmonary pressures compared with right heart catheterization despite acceptable average agreement [18, 19]. The implications for bedside interpretation are discussed under Comparison With Invasive Hemodynamics.

## 3. Echocardiographic Estimation of Mean and End‐Diastolic Pulmonary Artery Pressure

Continuous‐wave Doppler assessment of pulmonary regurgitation (PR) can provide complementary estimates of mean and end‐diastolic pulmonary artery pressure using the modified Bernoulli equation [20]. Peak early diastolic PR velocity reflects the pressure gradient between the pulmonary artery and RV during early diastole and may be used to estimate the mean pulmonary artery pressure. End‐diastolic PR velocity reflects the corresponding end‐diastolic pressure gradient and may be used to estimate pulmonary artery end‐diastolic pressure:

(4) Mean pulmonary artery pressure mmHg≈4×early diastolic PR velocity m/s2+RAP mmHg,

(5) Pulmonary artery end‐diastolic pressure mmHg≈4×end‐diastolic PR velocity m/s2+RAP mmHg.

Both equations use RAP as a practical approximation of the relevant RV diastolic pressure, which introduces additional uncertainty when RV filling pressures or relaxation are abnormal. The mean pulmonary artery pressure may also be estimated from PASP using the relationship described by Chemla et al. [21]:

(6) Mean pulmonary artery pressure mmHg≈0.612×PASP mmHg+.

A weighted estimate using systolic and end‐diastolic pulmonary artery pressures is also used [4]:

(7) Mean pulmonary artery pressure mmHg≈PASP+2×pulmonary artery end‐diastolic pressure3.

These methods provide complementary internal consistency checks rather than interchangeable substitutes for invasive catheter measurements. TEE‐derived early and end‐diastolic PR velocities obtained from the upper‐esophageal aortic arch short‐axis view are demonstrated in Figure 18, and corresponding TTE measurements from the parasternal RV outflow view are demonstrated in Figure 19.

**FIGURE 18:**
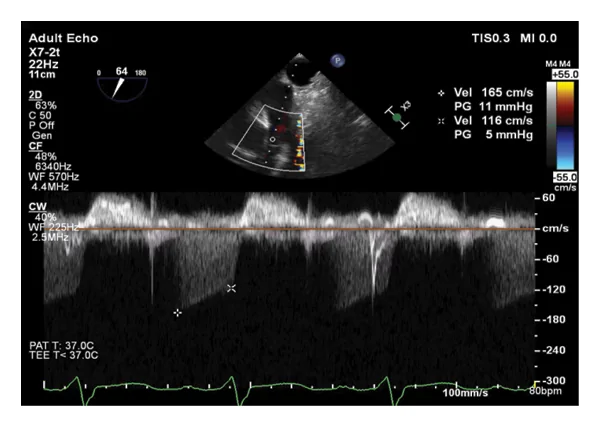
Transesophageal continuous‐wave Doppler recording of pulmonary regurgitation, demonstrating early and end‐diastolic velocities of 1.65 and 1.16 m/s, respectively. The corresponding pressure gradients are approximately 11 and 5 mmHg before addition of right atrial pressure.

**FIGURE 19:**
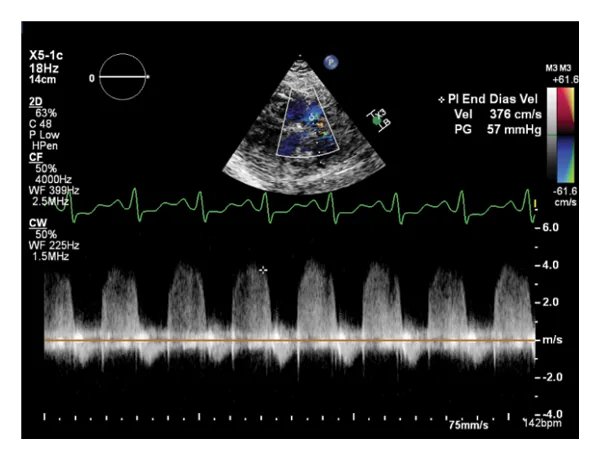
Transthoracic continuous‐wave Doppler recording of pulmonary regurgitation, demonstrating an early diastolic velocity of approximately 4.4 m/s and an end‐diastolic velocity of 3.76 m/s.

An additional empirical approach estimates the mean pulmonary artery pressure from the complete TR continuous‐wave Doppler envelope, assuming no RVOT obstruction. The entire envelope is traced, allowing echocardiographic software to calculate the mean systolic RV–RA pressure gradient by averaging the instantaneous pressure gradients throughout systole:

(8) Mean pulmonary artery pressure mmHg≈mean TR pressure gradient mmHg+RAP mmHg.

This less established approach requires optimal alignment and a complete, well‐defined TR envelope. In an ICU validation study, an adequate signal was obtainable in only 23 of 53 patients, and the limits of agreement with invasive mean pulmonary artery pressure were −7.9 to +11.6 mmHg [22]. PR Doppler has also undergone limited ICU‐specific validation. Its envelopes are frequently less distinct, and severe PR, rapid pressure equalization, arrhythmia, uncertain end‐diastolic timing, and uncertainty in RAP may substantially affect the estimates. These methods are therefore most useful as complementary components of the overall examination.

### 3.1. RV Outflow Tract Doppler

RVOT pulsed‐wave Doppler provides site‐specific assessment of blood‐flow velocity within a selected sample volume. In a normal, low‐resistance, highly compliant pulmonary vascular bed, flow accelerates smoothly, reaches peak velocity near mid‐systole, and then gradually decelerates. As pulmonary vascular impedance increases, the RV encounters greater opposition to ejection and reflected pressure waves return earlier during systole. Consequently, peak velocity occurs earlier, pulmonary artery acceleration time (PAAT) shortens, and more severe increases in impedance may produce midsystolic flow deceleration or notching.

The pulsed‐wave Doppler sample volume is generally placed in the RVOT immediately proximal to the pulmonic valve. PAAT is measured from the onset of systolic flow to peak velocity (Figure 20). Increasing pulmonary vascular load shifts the peak flow earlier in systole and shortens PAAT. In the original study by Dabestani et al., the following relationship was derived for PAAT ≤ 120 ms [23]:

(9) Mean pulmonary artery pressure mmHg≈90−0.62×PAAT ms.

**FIGURE 20:**
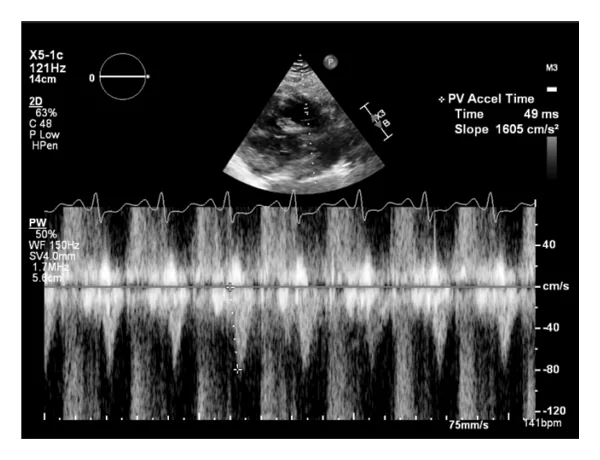
Transthoracic pulsed‐wave Doppler recording of pulmonary outflow, demonstrating a pulmonary artery acceleration time of 49 ms. Acceleration time is measured from flow onset to peak velocity.

Very short PAAT values should be interpreted primarily as evidence of abnormal pulmonary vascular load rather than as precise estimates of the mean pulmonary artery pressure. A heart‐rate‐corrected PAAT ≤ 90 ms identified PVR ≥ 3 Wood units with 84% sensitivity and 85% specificity in a simultaneous echocardiography and right heart catheterization study of 56 selected patients [24].

Heart rate, rhythm, RV contractility, preload, stroke volume, sampling position, and ventilatory conditions influence PAAT. A short PAAT may therefore reflect increased pulmonary vascular load, reduced RV ejection, or both. In an ICU sepsis cohort, PAAT was measurable in 81% of patients, including some without a measurable TR velocity, supporting its feasibility but not establishing definitive accuracy against invasive measurements [25].

Systolic notching of the RVOT envelope represents abrupt deceleration of forward flow caused by increased pulmonary vascular impedance and the early return of reflected pressure waves during RV ejection (Figure 21). Midsystolic notching supports the presence of abnormal pulmonary vascular load, particularly increased PVR and reduced pulmonary arterial compliance but is not disease‐specific. In a pulmonary hypertension referral cohort, midsystolic notching was 96% specific and 71% sensitive for PVR > 5 Wood units [26]. However, this diagnostic performance should not be extrapolated directly to unselected ICU populations.

**FIGURE 21:**
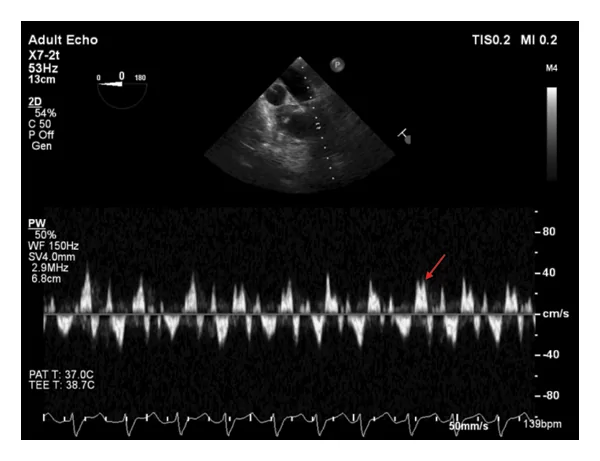
Transesophageal pulsed‐wave Doppler recording of pulmonary outflow, demonstrating midsystolic notching of the spectral envelope (red arrow).

RVOT VTI provides important supplemental information about RV forward stroke volume. It represents the stroke distance, defined as the distance traveled by blood during systolic ejection. Using the continuity principle, forward stroke volume and pulmonary blood flow are calculated as follows:

(10) RVOT cross‐sectional area cm2=π×RVOT diameter cm22,

(11) Forward stroke volume mL=RVOT cross–sectional area cm2×RVOT VTI cm,

(12) Pulmonary blood flow L/min=forward stroke volume mL×heart rate beats/min1000,.

In the absence of significant shunting or PR, pulmonary blood flow approximates effective cardiac output. Accurately measuring the RVOT diameter is often difficult, particularly in critically ill patients with suboptimal imaging windows. The RVOT is anteriorly positioned; its borders may be poorly defined, and its geometry is not perfectly circular. Because the radius is squared when calculating the cross‐sectional area, even a small error in RVOT diameter measurement can produce a substantially larger error in calculated stroke volume and cardiac output. An RVOT VTI > 18 cm is considered normal in the reference setting [4]. Given the limitations of RVOT area measurement, RVOT VTI alone is commonly used in critical care as a directional surrogate of RV forward stroke volume rather than as a precise measurement of cardiac output. Figure 22 illustrates a TEE pulmonary outflow recording from the proximal pulmonary artery; its sampling site should be distinguished from a proximal RVOT measurement.

**FIGURE 22:**
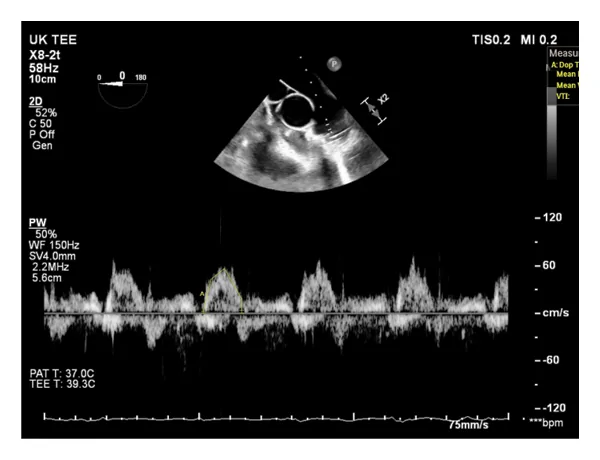
Transesophageal pulsed‐wave Doppler recording from the mid‐esophageal ascending aortic short‐axis view for assessment of pulmonary outflow velocity–time integral. The sample is positioned in the proximal pulmonary artery. The complete systolic envelope is traced to measure stroke distance; sampling sites should be consistent for serial comparisons.

Serial assessment of RVOT VTI can be used as a surrogate of changes in RV stroke volume and forward flow. A decrease in estimated PASP accompanied by an increase in RVOT VTI suggests a different physiologic response from a decrease in both measurements. The former may indicate reduced pulmonary vascular load with improved forward flow, whereas the latter may reflect worsening RV pump failure and diminished pressure‐generating capacity. This pressure‐flow integration is particularly useful during ventilator adjustment, pulmonary vasodilator therapy, fluid removal, or treatment of acute RV failure. However, RVOT VTI remains load‐dependent and is influenced by heart rate, rhythm, preload, severe TR, RV contractility, positive‐pressure ventilation, and rapidly changing hemodynamic conditions. Measurement accuracy also depends on appropriate Doppler alignment, consistent sample‐volume positioning, and a well‐defined spectral envelope. RVOT or pulmonic valve obstruction may compromise these calculations, and intracardiac shunting may cause pulmonary flow to differ from systemic cardiac output. Substantial PR may cause systolic forward flow to overestimate the effective net pulmonary output. Serial measurements should therefore be obtained from the same view and sampling location under comparable ventilatory and hemodynamic conditions.

### 3.2. Doppler Estimates of PVT

Invasive PVR is calculated from the transpulmonary pressure gradient and cardiac output:

(13) PVR Wood units=mean pulmonary artery pressure mmHg−PAWP mmHgcardiac output L/min.

Doppler equations estimate PVR empirically without directly measuring PAWP or cardiac output. Their input variables therefore cannot be assumed to reproduce the components of the invasive calculation.

The original Doppler equation described by Abbas et al. is [27]:

(14) Estimated PVR Wood units≈100.16×VTRRVOT VTI+.

Here, VTR is expressed in m/s and RVOT VTI in cm. A VTR/RVOT VTI ratio > 0.275 suggests that PVR is likely > 6 Wood units. For higher resistance ranges, the following equation has been proposed [28]:

(15) Estimated PVR Wood units≈5.190.4×VTR2RVOT VTI−.

In the subsequent pooled analysis of five validation cohorts comprising 150 patients, Abbas et al. found that the VTR/RVOT VTI ratio correlated with catheter‐derived PVR at *r* = 0.76, whereas the squared formulation correlated slightly more strongly at *r* = 0.79 and performed better at PVR > 6 Wood units [28]. Both equations inherit the technical limitations of TR velocity and RVOT VTI. Small TR velocity errors are magnified by the squared‐velocity formulation, whereas a low RVOT VTI caused by reduced preload, arrhythmia, severe TR, or RV pump failure may falsely increase estimated PVR. These equations were validated in small, selected, predominantly non‐ICU cohorts and may have limited precision in individual critically ill patients. They are therefore best used as supportive components of hemodynamic phenotyping rather than as substitutes for invasively measured PVR; their performance for tracking treatment‐related changes in the ICU remains uncertain.

### 3.3. RV–PA Coupling

TAPSE/PASP is the most widely used echocardiographic surrogate of RV–PA coupling:

(16) TAPSEPASP=TAPSE mmPASP mmHg.

Longitudinal RV systolic performance is interpreted relative to the pressure against which the RV ejects. Thus, an apparently normal TAPSE may still represent inadequate contractile adaptation when PASP is markedly elevated.

In 52 patients with severe pulmonary hypertension, a TAPSE/PASP threshold of approximately 0.31 mm/mmHg discriminated invasive uncoupling with 87.5% sensitivity and 75.9% specificity [29]. A systematic review found only moderate correlation between TAPSE/PASP and pressure–volume loop‐derived Ees/Ea [30]. The ratio is therefore a surrogate rather than a noninvasive measurement of coupling.

ICU data support prognostic relevance but do not establish a universal threshold. In 118 mechanically ventilated patients with septic shock, TAPSE/PASP was associated with ICU and 1‐year mortality, with an empirically derived threshold of approximately 0.50 mm/mmHg [31]. In another sepsis cohort, 106 of 131 patients had analyzable RVOT Doppler. Among these 106 patients, TAPSE/PASP was measurable in 77 (73%), of whom 15 (19%) had values < 0.31 mm/mmHg [25]. In 1077 critically ill patients, lower TAPSE/TR velocity ratios were independently associated with ICU mortality, particularly in respiratory and septic subgroups [32]. Differences in study population, mechanical ventilation, RAP estimation, and the denominator used prevent direct equivalence among these thresholds.

TAPSE/PASP should be interpreted with RV size, septal flattening, FAC, S′, RV free‐wall strain, three‐dimensional RVEF when available, RVOT VTI, and clinical evidence of venous congestion or low output. No universal ICU coupling thresholds have been established for alternative ratios based on FAC, strain, S′, or three‐dimensional RVEF. These measurements strengthen characterization of RV performance but do not convert an echocardiographic ratio into invasive Ees/Ea. A summary of echocardiographic assessment of RV function, estimated pulmonary artery pressures, and afterload is presented in Table 3.

**TABLE 3:** Echocardiographic methods for evaluating RV function, pulmonary artery pressures, PVR, and RV–PA coupling.

Parameter	Acquisition	Interpretation	Limitations
RAP	IVC size and collapse or measured pressure	Use measured RAP or CVP when reliable; fixed IVC categories are least reliable during positive‐pressure ventilation	Ventilation, intra‐abdominal pressure, spontaneous effort, venous cannulation
PASP	4 × VTR^2^ + RAP (velocity in m/s; pressure in mmHg)	High PASP requires RV pressure‐generating capacity; falling PASP may reflect unloading or deteriorating RV output	Alignment, envelope quality, RAP error, severe TR, low‐flow RV failure
Mean and end‐diastolic pulmonary artery pressure	Early and end‐diastolic PR Doppler; convert velocity to a pressure gradient before adding RAP	Useful for internal consistency	PR quality, timing, RAP error
PAAT	Onset of RVOT flow to peak velocity	Marked shortening supports abnormal loading; interpret with RVOT VTI	Heart rate, output, RV function, ventilation
RVOT notching	RVOT spectral morphology	Midsystolic notching supports abnormal pulmonary vascular load	Signal quality; primarily validated in pulmonary hypertension cohorts
RVOT VTI	Pulsed‐wave Doppler proximal to the pulmonic valve	Important companion to pressure measurements	Preload, rhythm, contractility, sampling position
PVR estimate	TR velocity/RVOT VTI equations	Supports physiologic phenotyping; ICU accuracy and performance for tracking treatment response remain uncertain	Does not measure PAWP or cardiac output; confounded by low‐flow states
TAPSE and S′	Longitudinal displacement and velocity	Simple and reproducible but incompletely represent global RV function	Load, Doppler angle, surgery, pacing, TR, and regional motion
FAC	Two‐dimensional end‐diastolic and end‐systolic area change	Useful when longitudinal indices are misleading	Image quality, exclusion of the RVOT, and single‐plane assessment
RV free‐wall strain	Speckle‐tracking deformation	Sensitive to early and regional dysfunction; interpret relative to loading conditions	Software, tracking quality, and load dependence
Three‐dimensional RVEF	Three‐dimensional RV volumes and ejection fraction	Integrates longitudinal and nonlongitudinal contraction and provides comprehensive RV volumes and global ejection fraction when acquisition is adequate	Acquisition completeness, resolution, stitching artifacts, software, and limited ICU availability
RV dP/dt	Time for TR velocity to increase from 1 to 2 m/s	Adjunctive surrogate of RV contractile performance	Requires measurable TR and optimal continuous‐wave Doppler alignment; load dependent and unreliable with severe TR
TAPSE/PASP	TAPSE in mm divided by PASP in mmHg	Potential coupling marker; no universal ICU threshold	Inherits errors from both variables and remains population dependent

### 3.4. Comparison With Invasive Hemodynamics

Right heart catheterization remains the reference standard for hemodynamic classification of pulmonary hypertension and calculation of PVR [33]. Doppler echocardiography derives pressure differences from velocity and adds estimated RAP, whereas catheterization measures absolute intravascular pressure referenced to an external zero. Both methods are sensitive to respiratory phase and intrathoracic pressure, particularly during positive‐pressure ventilation.

Validation studies illustrate the distinction between population‐level accuracy and individual precision. Fisher et al. reported minimal average bias for estimated PASP, but 95% limits of agreement with catheter measurements ranged from −40.0 to +38.8 mmHg [18]. D’Alto et al. similarly found little average bias for mean pulmonary artery pressure, with limits of agreement of approximately −18 to +19 mmHg [19]. These are different pressure measurements, but both studies demonstrate that acceptable average agreement does not ensure reliable estimates in an individual patient. Discordance may increase when studies are separated in time or when ventilation, volume status, pulmonary vascular tone, or cardiac output changes between examinations.

When both modalities are available, the comparison should begin with quality control. Catheter leveling and zeroing, waveform quality, respiratory timing, and the Doppler envelope should be verified. Invasively measured RAP should be used in the Doppler equation whenever feasible, and values should be compared under the same ventilatory and hemodynamic conditions. Persistent disagreement should prompt reassessment rather than averaging the numbers. Echocardiography adds information that a pressure tracing cannot provide, including RV morphology, septal interaction, valvular pathology, and the visible response of the RV to load.

## 4. Practical Integrated Approach in the ICU

A focused ICU examination can be organized as follows:

1. Record heart rate, rhythm, blood pressure, ventilatory mode, PEEP, oxygenation, and vasoactive support.
2. Estimate RAP from the IVC only when the physiologic setting is appropriate and use a reliable directly measured RAP when available. In the ICU, central venous pressure (CVP) obtained through a central venous catheter can often be used as a surrogate for RAP.
3. Acquire the best TR envelope from multiple windows and calculate PASP only when the signal and assumptions are acceptable.
4. Obtain PR velocities, PAAT, RVOT VTI, and RVOT envelope morphology when technically feasible to provide complementary pressure, flow, and impedance information.
5. Assess RV size and systolic function, then interpret TAPSE/PASP only when both components are credible.
6. Reconcile the measurements internally. A very high calculated PASP with normal RV morphology and normal RVOT flow, or a low PASP with severe RV dilation and low forward flow, should trigger a review of signal quality and physiology.
7. Proceed to or rely on invasive hemodynamics when precise classification, PAWP, cardiac output, or PVR will change management.

### 4.1. Clinical Integration in Common ICU Phenotypes

#### 4.1.1. ARDS and Positive‐Pressure Ventilation

Acute cor pulmonale was identified in approximately 22% of patients with moderate to severe ARDS in two prospective cohorts [34, 35]. ARDS and positive‐pressure ventilation may increase RV afterload through pulmonary vascular injury, hypoxemia, hypercapnia, acidemia, and alveolar overdistension, while elevated intrathoracic pressure may simultaneously reduce venous return and RV preload [36]. Some of the beneficial hemodynamic effects of prone positioning may be mediated by RV unloading. Improved oxygenation, more homogeneous lung recruitment, reduced hypercapnia, and less regional overdistension may decrease PVR and pulmonary vascular impedance, thereby reducing RV dilation and septal dyskinesia [37].

Bedside echocardiography has an important role in detecting and monitoring these cardiopulmonary interactions. Serial assessment of RV size and systolic function, septal configuration, pulmonary artery pressures, and forward flow may identify acute cor pulmonale and demonstrate the hemodynamic response to changes in PEEP, recruitment, or prone positioning. Lazzeri et al. emphasized the complementary roles of TTE and TEE in recognizing the continuum of pulmonary vascular and RV abnormalities in ARDS and individualizing ventilatory management [36]. Ajam et al. specifically reviewed echocardiography in prone patients with ARDS, highlighting the feasibility of TTE, the complementary role of TEE when transthoracic imaging is inadequate, and the value of echocardiography for assessing cardiopulmonary responses to prone positioning [38].

### 4.2. Sepsis and Septic Shock

Sepsis may impair both components of the RV–PA interaction. Direct septic myocardial depression may reduce RV contractility, while pulmonary endothelial dysfunction, microvascular obstruction, hypoxemia, hypercapnia, acidemia, concomitant ARDS, and positive‐pressure ventilation may increase PVR and pulmonary arterial impedance. Vasodilation, fluid administration, PEEP, and vasoactive therapy may simultaneously alter RV preload. In 393 patients evaluated early during sepsis or septic shock, RV dysfunction was present in approximately 48% and was independently associated with higher odds of 28‐day mortality (adjusted odds ratio 3.4; 95% confidence interval 1.7–6.8) [39].

Echocardiography can help determine whether the predominant abnormality is increased pulmonary vascular load, impaired RV myocardial performance, inadequate preload, or combined RV failure. TR‐ and PR‐derived pressures, PAAT, RVOT systolic notching, and systolic septal flattening provide evidence of increased pulmonary vascular load. RV size, TAPSE, S′, FAC, RV free‐wall strain, and three‐dimensional RVEF characterize the RV response to that load, while TAPSE/PASP provides a surrogate of RV–PA coupling. RVOT VTI assesses forward flow, and the IVC, measured CVP, TR severity, and other evidence of venous congestion help define the hemodynamic consequences.

The integrated pattern is more informative than any individual measurement. Short PAAT, RVOT notching, and systolic septal flattening with preserved RV systolic function and RVOT VTI suggest increased pulmonary vascular load with preserved adaptation. Reduced systolic indices without clear pressure‐overload findings may suggest predominant septic myocardial dysfunction. A small, underfilled RV with low RVOT VTI and no venous congestion instead supports inadequate preload. RV dilation accompanied by systolic septal flattening, reduced systolic function, low RVOT VTI, and venous congestion suggests combined increased afterload and RV pump failure. A declining PASP accompanied by worsening RV dilation and decreasing RVOT VTI may reflect loss of RV pressure‐generating capacity rather than improvement.

Sepsis‐specific studies support the feasibility and prognostic relevance of pulmonary artery Doppler measurements and RV–PA coupling indices, but they remain observational and do not establish treatment thresholds [25, 31, 32, 40]. In 282 mechanically ventilated patients with septic shock, RV failure defined by RV dilation and CVP ≥ 8 mmHg occurred in 42% and was associated with reduced fluid responsiveness. TAPSE remained normal in 63.5% of these patients, emphasizing the need for multiparametric assessment [41]. Serial echocardiography can subsequently determine whether ventilator adjustment, correction of gas‐exchange abnormalities, fluid management, or vasoactive therapy improves RV loading and forward flow.

### 4.3. Acute Pulmonary Embolism and Decompensated Pulmonary Hypertension

Acute pulmonary embolism abruptly increases PVR and RV afterload. Because the nonhypertrophied RV has limited capacity to generate high systolic pressure, acute RV dilation, septal flattening, reduced LV preload, and circulatory failure may occur despite only moderately elevated PASP. In contrast, chronic pulmonary hypertension produces progressive RV hypertrophy and remodeling, allowing the adapted RV to generate substantially higher pulmonary artery pressures. Decompensation occurs when this adaptation fails, resulting in RV dilation, worsening TR, reduced forward flow, and systemic venous congestion. Thus, the same PASP may have markedly different implications depending on RV wall thickness, chamber geometry, contraction, and RVOT VTI. RV hypertrophy and marked RA enlargement favor chronic pressure overload, although acute‐on‐chronic disease may blur this distinction.

In suspected acute pulmonary embolism, echocardiography should evaluate RV size, systolic function, septal configuration, TR velocity, PAAT, RVOT flow, and the presence of right‐heart thrombus. Echocardiography cannot exclude pulmonary embolism in a hemodynamically stable patient. However, in an unstable patient who cannot safely undergo computed tomography, unequivocal RV pressure overload without another explanation may support urgent reperfusion therapy [42].

The 60/60 sign combines a PAAT ≤ 60 ms with a peak systolic TR pressure gradient ≤ 60 mmHg. It reflects early peaking of RV outflow caused by an abrupt increase in pulmonary vascular impedance despite the limited pressure‐generating capacity of the acutely overloaded RV. The original study reported a sensitivity of 25% and a specificity of 94% [43]. Therefore, its presence supports acute pulmonary embolism, but its absence does not exclude the diagnosis, and false‐positive findings may occur in other causes of acute pulmonary vascular loading.

McConnell sign consists of akinesis or severe hypokinesis of the mid‐RV free wall with preserved apical contraction (Supporting Video 1). The original study reported 77% sensitivity and 94% specificity for acute pulmonary embolism [44]. However, a subsequent study found the sign in 70% of patients with acute pulmonary embolism and 67% with RV infarction, yielding a specificity of only 33% in that selected comparison [45]. McConnell sign is therefore less specific than originally believed and should not be interpreted in isolation. Serial echocardiography can subsequently assess changes in RV size, septal flattening, systolic function, and forward flow following reperfusion or treatment of decompensated pulmonary hypertension.

### 4.4. Left‐Sided Heart Disease and Postcapillary Pulmonary Hypertension

Postcapillary pulmonary hypertension commonly results from LV systolic or diastolic dysfunction, mitral or aortic valve disease, or other conditions that increase left atrial (LA) pressure. It is defined invasively by a mean pulmonary artery pressure > 20 mmHg and PAWP > 15 mmHg. PVR ≤ 2 Wood units indicates isolated postcapillary pulmonary hypertension, whereas PVR > 2 Wood units identifies combined postcapillary and precapillary pulmonary hypertension, in which pulmonary vasoconstriction or vascular remodeling has added a precapillary component [33].

Echocardiography can identify the underlying left‐sided pathology and estimate the probability of elevated LA pressure by integrating LV systolic function, mitral valve disease, transmitral inflow, mitral annular early diastolic velocity (e′), the ratio of transmitral early diastolic velocity to e′ (E/e′), LA size and strain, pulmonary venous flow, and lung ultrasound evidence of pulmonary congestion [46]. LA enlargement generally reflects the chronicity of elevated filling pressure and may be absent when LA pressure rises acutely. No single parameter should be interpreted in isolation, particularly in patients with atrial fibrillation, tachycardia, significant mitral valve disease, mitral annular calcification, pacing, mechanical ventilation, or rapidly changing loading conditions.

A high PASP accompanied by evidence of elevated LA pressure, preserved RVOT VTI, and relatively preserved PAAT without systolic notching supports predominantly passive transmission of left‐sided pressure. In contrast, high PASP accompanied by a short PAAT, RVOT systolic notching, reduced RVOT VTI, RV hypertrophy or dilation, septal flattening, and impaired RV systolic function raises concern for a superimposed precapillary component and progressive RV–PA uncoupling.

These findings provide physiologic phenotyping but do not establish PAWP or distinguish isolated from combined postcapillary pulmonary hypertension with sufficient precision. In 98 mechanically ventilated critically ill patients, application of the 2016 echocardiographic diastolic assessment algorithm was indeterminate in 48 patients (49%); among the 50 classifiable examinations, sensitivity and specificity for detecting PAWP ≥ 18 mmHg were both 74% [47]. These findings should not be interpreted as a validation of the updated 2025 recommendations. Doppler‐derived PVR and estimates of LA pressure cannot replace invasive measurement when PAWP, cardiac output, or PVR will alter diagnosis or management.

Evidence from heart failure populations provides additional context for TAPSE/PASP. In 293 patients with heart failure, predominantly with reduced LV ejection fraction, Guazzi et al. found that the ratio improved prognostic assessment compared with either measurement considered separately; values < 0.36 mm/mmHg were associated with worse outcomes [48]. In 97 patients with heart failure with preserved ejection fraction, Gorter et al. associated a reduced ratio with a precapillary component of pulmonary hypertension and increased mortality [49]. These observations support integrating RV function with pulmonary pressure for phenotyping and risk stratification. However, the cohorts were selected, and the latter study used earlier hemodynamic definitions. Their findings do not establish a universal ICU treatment threshold or replace invasive classification.

### 4.5. Evidence Gaps and Future Directions

Most equations for PAAT, Doppler‐derived PVR, and TAPSE/PASP were developed in stable cardiology or pulmonary hypertension populations rather than in mechanically ventilated critically ill patients. Dynamic changes in pleural pressure may affect both the numerator and denominator of coupling ratios, further complicating their interpretation. ICU‐specific thresholds for RV–PA uncoupling remain inconsistent, and values derived from chronic pulmonary hypertension populations cannot be reliably extrapolated to sepsis, ARDS, acute pulmonary embolism, or postcardiotomy RV dysfunction. Severe TR, cardiac surgery, pacing, regional RV dysfunction, and discordance between longitudinal and nonlongitudinal contraction further limit the reliability of TAPSE‐based coupling indices.

It also remains uncertain whether serial changes in Doppler‐derived afterload or coupling indices reliably identify therapeutic responsiveness or improve clinical outcomes. Future prospective studies should prioritize simultaneous echocardiographic and invasive hemodynamic measurements obtained under standardized ventilatory and hemodynamic conditions. These studies should integrate pressure, flow, RV geometry, and contraction patterns rather than evaluate isolated measurements; establish disease‐specific ICU reference ranges and thresholds; and determine whether intervention‐related changes predict meaningful hemodynamic responses and patient‐centered outcomes.

## 5. Conclusion

Echocardiography can characterize RV afterload and RV–PA coupling more comprehensively than a single PASP estimate. TR and PR Doppler and IVC assessment provide estimates of right‐sided pressures; RVOT acceleration time and systolic notching reflect pulmonary vascular impedance; RVOT VTI provides a surrogate of forward flow; and TAPSE/PASP relates RV systolic performance to the pressure against which it ejects. Their value is greatest when measurements are acquired carefully, interpreted collectively, and integrated with RV geometry, systolic function, and the patient’s ventilatory and hemodynamic state.

Despite its limitations, echocardiography remains an essential diagnostic adjunct because of its bedside availability, noninvasive transthoracic assessment and complementary TEE imaging, and capacity for repeated dynamic surveillance during therapeutic interventions. Invasive hemodynamics remain necessary when precise pulmonary artery pressures, PAWP, cardiac output, or PVR measurements are required to establish the diagnosis or guide management. Accordingly, echocardiography and invasive monitoring should be regarded as complementary rather than competing methods of hemodynamic assessment.

## Funding

The author received no financial support for the research, authorship, and/or publication of this article.

## Conflicts of Interest

The author declares no conflicts of interest.

## Supporting Information

Additional supporting information can be found online in the Supporting Information section.

## Supporting information


**Supporting Information** Video 1. Right ventricular (RV)‐focused apical four‐chamber transthoracic echocardiographic view demonstrating preserved apical contraction with severe hypokinesis of the RV free wall, consistent with McConnell sign in a patient with acute pulmonary embolism.

## Data Availability

This narrative review synthesizes published literature. No new research datasets were generated or analyzed.
